# Peer rejection and internet gaming disorder: the mediating role of relative deprivation and the moderating role of grit

**DOI:** 10.3389/fpsyg.2024.1415666

**Published:** 2025-01-15

**Authors:** Jingjing Li, Chang Wei, Jiachen Lu

**Affiliations:** ^1^School of Health Management, Guangzhou Medical University, Guangzhou, China; ^2^Guangzhou Maritime College, Guangzhou, China; ^3^School of Education, Research Center of Rural Education and Cultural Development of the Key Research Institute of Humanities and Social Sciences in Hubei Province, Hubei University of Science and Technology, Xianning, China

**Keywords:** internet gaming disorder, relative deprivation, peer rejection, grit, addiction

## Abstract

**Background:**

Internet Gaming Disorder (IGD) is a new behavioral addiction. A large number of empirical studies have shown that Internet Gaming Disorder has a high level of comorbidity with other diseases, including depression, anxiety, obesity, internalizing and externalizing behavioral problems, however, little is known about the mediating and moderating mechanisms underlying this relation. The current study adopted a three-time longitudinal study investing the mediating effect of relative deprivation on the association between peer rejection and IGD, and whether this mediating effect was moderated by the grit.

**Methods:**

A total of 1,065 students in China anonymously completed three-time longitudinal study questionnaires. The average age was 10.19 years (SD = 0.75) and the interval between measurements was 6 months.PROCESS for SPSS proposed by Hayes was used to test a moderated mediation model, with gender, age as covariates.

**Results:**

T1 peer rejection positively predicted T3 Internet Gaming Disorder. Relative deprivation at T2 plays a complete mediating role between peer rejection at T1 and Internet gaming disorder at T3. At the same time, it was found that the personality trait of T3 grit plays a moderating role in the relationship between T2 relative deprivation and T3 Internet gaming disorder. This suggests that peer rejection is an important predictor of Internet Gaming Disorder, and that individuals with high levels of grit are less likely to become addicted to Internet games even if they experience relative deprivation.

**Limitations:**

Measures of study variables were self-reported. Affected by factors such as social desirability, the research results may be biased.

**Conclusion:**

These findings emphasize relative deprivation as a potential mechanism linking peer rejection IGD. Grit was an important protective factor to weaken this indirect effect. Intervention programs aimed at reducing IGD may benefit from the current research.

## Introduction

Internet Gaming Disorder is a new behavioral addiction. According to the Diagnostic and Statistical Manual of Mental Disorders fifth edition (DSM-5), Internet Gaming Disorder (IGD) is defined as “persistent and repeated use of the Internet to engage in gaming, usually with other players, resulting in clinically significant injury or pain.” Following the American Psychiatric Association (APA), the World Health Organization also included “gaming disorder” as a mental illness in the International Classification of Diseases (ICD-11) in 2019 (Liu et al., 2022). A large number of empirical studies have shown that Internet Gaming Disorder has a high level of comorbidity with other diseases, including depression, anxiety, obesity, internalizing and externalizing behavioral problems, alexithymia, ADHD, and obsessive-compulsive disorder (Lozano-Blasco et al., 2022; Yang et al., 2022; Zhai et al., 2020). Most previous studies have explored the mechanisms of online game addiction from the perspective of individual factors, such as self-control (Kim and Choi, 2020), emotional regulation (Tripp and Mcdevitt-Murphy, 2015), self -esteem (Ayas and Horzum, 2013), sensation seeking (Kim and Choi, 2020; Tian et al., 2018). However, from the perspective of social situations (such as companions), the influence mechanism of Internet Gaming Disorder is revealed. The compensatory network usage model suggests that (Kardefelt-Winther, 2014), when individuals are in unfavorable situations, they tend to escape reality through online activities and make up for the shortcomings in real life through online activities. The greater the external pressure an individual experiences, the longer they spend on online activities, thus forming a vicious cycle. Peer rejection is an unfavorable factor for individual development (Badenes-Ribera et al., 2019), and will drive individuals to immerse themselves in online games (Poon, 2018), but it is still unknown through what mechanism peer rejection affects online game addiction.

### Peer rejection and internet gaming disorder

**Figure 1:**
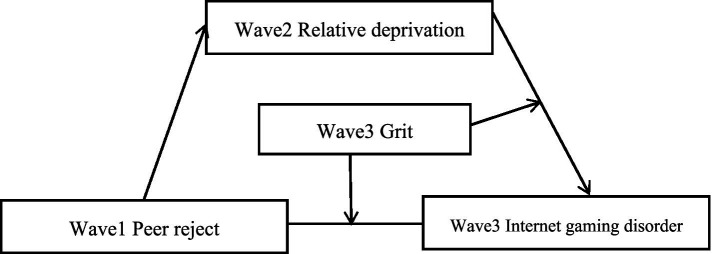
The proposed moderated mediation model.

Peer rejection refers to the various forms of rejection an individual encounters from peers in daily life, such as indifference, isolation, discrimination, rejection, and exclusion (Bierman, 2004), which is a negative interpersonal experience. According to the need-threat time model, peer rejection can easily bring many negative emotions to individuals (Leary, 1990) and cause social pain (Eisenberger and Lieberman, 2004). Individuals whose long-term needs are not met may enter the withdrawal stage, and try to escape from real life (Ren et al., 2016). Peer rejection will damage an individual’s sense of security, belonging, self-esteem and other related psychological needs, thereby causing individuals to become addicted to online games (Badenes-Ribera et al., 2019; Xin et al., 2021) (Figure 1).

Although individuals can temporarily evade real life through online games, they also increase the risk of Internet Gaming Disorder. According to Compensation network use models (Kardefelt-Winther, 2014), when individuals are in negative situations (such as companion exclusion), it is easy to make up for the psychological needs in real life through online games. The greater the pressure, the longer the time of the individual spends on online games, thus the cycle of evil, and eventually leads to online game addiction. Researchers found that the exclusive experience will increase the individual’s network addiction behavior (Badenes-Ribera et al., 2019; Poon, 2018) and also found that companion rejection is a social risk factors that increase the use of individual social media. Individuals who are more severely rejected by their peers are more addicted to social media use (He et al., 2024). Based on this, it is speculated that peer rejection can positively predict individual online game addiction.

### The mediating role of relative deprivation

Relatively deprivation refers to a subjective cognition that individuals or groups and groups perceive their status and situation, and then experience the deprivation of basic power (Mummendey et al., 1999; Zhang and Tao, 2013). According to the classical theory of relative deprivation (Mummendey et al., 1999), individuals in disadvantaged situations (such as those who are rejected by their peers) may experience a sense of deprivation of basic rights during the social comparison process. This sense of deprivation can easily have a negative impact on their psychological and behavioral adaptation and induce problematic behaviors.

This study believes that relative deprivation plays a mediating role between peer rejection and Internet game addiction, mainly for the following reasons:

First, peer rejection triggers relative deprivation. Research has found that encountering rejection is closely related to the sense of relative deprivation. The more rejection one experiences, the stronger the sense of relative deprivation an individual perceives (Huang and Liu, 2021). Peer rejection can cause individuals to have negative emotions such as anger and dissatisfaction, and these negative emotions are important emotional components of relative deprivation (Crosby, 1976; Walker and Pettigrew, 1984; Smith et al., 2012). Peer rejection is a negative interpersonal experience that leads to a break in the individual’s connection with society, which in turn leads to the individual’s perception of deprivation.

Secondly, the sense of relative deprivation may cause online game addiction. Research has also pointed out that relative deprivation can lead individuals to develop a variety of negative problematic behaviors, such as substance abuse and Internet addiction (Osborne et al., 2012; Smith et al., 2012). As a negative subjective experience, the sense of relative deprivation strengthens an individual’s evaluation of being deprived of his rights and low social status, which can easily lead to frustration and depression, thereby increasing the risk of problematic behaviors (Callan et al., 2015). According to Cognitive-Behavior Model (Davis, 2001), an individual’s non-adaptive cognitive belief in the world has led to its considering that the online world can meet daily psychological needs more than the real world, and therefore appear online game addiction. Therefore, when an individual is in a deprived atmosphere for a long time, it will inevitably produce this unreasonable cognitive belief. In order to alleviate this discomfort, individuals can easily get instant satisfaction through online games. And online games have a fast and addicted effect, which may lead to Internet Gaming Disorder (Callan et al., 2011). Based on this, it is speculated that peer rejection indirectly affects Internet Gaming Disorder through relative deprivation.

### The moderating role of grit

Although peer rejection and relative deprivation both have an impact on Internet gaming addiction, there may be individual differences in this impact. Some psychological protective factors can have a positive effect, regulating and buffering the impact of risk factors on Internet Gaming Disorder. Grit is an important positive psychological trait, which is reflected in the individual’s continued interest and persistence in pursuing long-term goals even in the face of major obstacles. It is a personality trait that includes self-motivation, self-discipline and self-adjustment (Duckworth et al., 2007). Self-regulation theory believes that positive self-regulation characteristics allow individuals to continuously adjust themselves in the process of pursuing long-term goals, continue to make efforts when dealing with challenges, and actively seek help from various resources, thereby achieving behavioral changes (Abraham and Sheeran, 2000). Individuals with grit will show higher persistence when dealing with difficult situations (Lucas et al., 2015), and have a higher sense of self-efficacy in the face of stress (Jiang et al., 2021), and know how to use emotion regulation strategies to reduce the negative effects of stress and difficulties (Kalia et al., 2022), thereby maintaining positive emotions and reducing the risk of problem behaviors (Datu et al., 2018). Studies have shown that grit plays a moderating role between negative life events and problematic behaviors, and high levels of grit significantly weaken the impact of negative life events on suicidal ideation (Blalock et al., 2015; Pennings et al., 2015). From the perspective of resource theory, individual perseverance can also be regarded as an important positive psychological resource for individuals to cope with difficulties (such as peer rejection experiencing relative deprivation) (Hobfoll, 2011). People with grit are more likely to view difficulties as an inevitable part of growing up, which may make them less prone to problem behaviors (Datu et al., 2017). At the same time, people with high levels of grit will experience difficulties with a positive attitude and achieve self-regulation (Wolters and Hussain, 2015). It is speculated that grit plays a moderating role in the process of relative deprivation affecting Internet Gaming Disorder.

### The present study

Internet Gaming Disorder is the current important social realistic issue, and it is also a hot topic in academic research in the development of psychology, clinical psychology, social psychology, and psychological pathology. Addiction to internet game will seriously hinder individual physical and mental health. Therefore, in order to formulate a scientific and effective intervention plan, it is very necessary to clarify the reasons for Internet Gaming Disorder. However, most of the existing research is horizontal research, which cannot reveal the causal relationship between Internet Gaming Disorder and related psychological factors, and lacks long -term vertical research. Internet Gaming Disorder, a highly developmental problem, only through longer-term longitudinal research can the dynamic development process of Internet game addiction be revealed. Such research will have more reference value for intervention practice. Based on this, this study adopts a three-time longitudinal study and puts forward the following hypotheses:

Hypothesis 1: Peer rejection at T1 significantly and positively predicts online game addiction at T3.

Hypothesis 2: Relative deprivation at T2 plays a mediating role between peer rejection at T1 and online game addiction at T3.

Hypothesis 3: T3 grit plays a moderating role between T2 relative deprivation and T3 online game addiction.

## Methods

### Participants

Using the cluster sampling method, a total of 1,065 students from two primary schools in China. The interval between measurements was 6 months. In the first measurement, 1,065 subjects were 574 boys and 491 girls. The average age was 10.19 years (SD = 0.75). Among the 1,065 primary school students, 587 are in grade 4, accounting for 55.1%; and 478 are in grade 5, accounting for 44.9%. In the second measurement, 1,000 subjects were tested (537 boys and 463 girls). In the third measurement, 930 subjects were tested (477 boys and 453 girls). This study obtained informed consent from the children’s parents, who provided written informed consent, and the children provided written assent.

### Statistical analyses

We used SPSS 21.0 to examine descriptive statistics and correlations. We adopted Model 4 of PROCESS for SPSS proposed by Hayes to explore whether relative deprivation plays a mediating role between peer reject and Internet gaming disorder. To further test the moderating effect of the grit on the relationship between peer reject, relative deprivation and Internet gaming disorder, Model 15 of PROCESS for SPSS proposed by Hayes was used for data processing.

### Measures

#### Peer rejection

Peer rejection was measured using the social rejection scale developed by Thau et al. (2015), and later adapted by Xu and Niu (2019). The revised scale has a total of 6 items, using a 5-point Likert scoring method, 1 = “Completely disagree,” 5 = “completely agree,” calculate the average score, the higher the score, the more serious the rejection by peers. Cronbach’s alpha in this study was 0.92.

#### Relative deprivation

This study uses the Relative Deprivation Scale developed by Tian et al. (2021), which contains 10 items in total and is scored on a 5-point scale, 1 = strongly disagree, 5 = strongly agree, and the average score is calculated. The higher the score, the higher the relative deprivation. Feeling serious. Cronbach’s alpha in this study was 0.88 (T1) and 0.92 (T3).

#### Grift

This study used the Grit-S Grit Scale (Grit-S; Duckworth and Quinn, 2009). A total of eight items were scored on a five-point Likert scale. Participants were asked to range from 1 “Not like me at all” to 5 “Choose the option that best suits your situation from the five options “very much like me,” and calculate the average score. The higher the score, the higher the degree of perseverance. Cronbach’s alpha in this study was 0.78.

#### Internet gaming disorder

This study used the Internet Game Addiction Scale compiled by Pontes and Griffiths (2015) based on the diagnostic criteria of Internet Game Addiction in DSM-5. The scale consists of 9 items, requiring participants to report the frequency of IGD symptoms in the past 6 months. A 5-point rating scale is used, 1 (never), 2 (rarely), 3 (sometimes), 4 (often) and 5 (often), and the average score is calculated. The higher the score, the stronger the IGD tendency of the adolescent. Cronbach’s alpha in this study was 0.83 (T1) and 0.85 (T3).

## Results

We will present the results in three parts, and the flow chart is as follows: descriptive and correlation analysis (the relationship between variables) - mediation model analysis (the mediating effect of relative deprivation) - moderated mediation model analysis (the mediating effect of grit).

### Preliminary analyses

Descriptive statistics and Pearson correlation were used for descriptive and correlation analyses. The means, standard deviations, and correlation coefficients for all research variables are displayed in Table 1. Among the 1,065 students, 574 were boys, accounting for 53.9%, and 491 were girls, accounting for 46.1%. There were 587 students in the fourth grade, accounting for 55.1%, and 478 students in the fifth grade, accounting for 44.9%. In addition, there were 128 only children, accounting for 12.0%. A total of 607 students were from rural areas, accounting for 57.0%.

**Table 1:** Descriptive statistics and correlations for all variables.

Variable	1	2	3	4	5	6	7	8
1.Gender	1.00							
2.Age	0.12**	1.00						
3.Peer reject	−0.10**	0.03	1.00					
4.Relative deprivation at Wave 1	−0.01	0.06*	0.41***	1.00				
5.Relative deprivation at Wave 2	−0.02	0.07*	0.31***	0.38***	1.00			
6.Internet gaming disorder at Wave 1	0.21***	0.13***	0.25***	0.25***	0.18***	1.00		
7.Internet gaming disorder at Wave 3	0.15***	0.04	0.20***	0.18***	0.26***	0.48***	1.00	
8.Grit at Wave 3	0.00	−0.03	−0.23***	−0.20***	−0.31***	−0.21***	−0.29***	1.00
*Mean*	0.54	10.19	1.83	2.11	2.20	1.29	1.21	3.57
*SD*	0.50	0.75	0.99	0.56	0.90	0.41	0.38	0.69

Gender was dummy coded as 1 = male, 0 = female. *p < 0.05, **p < 0.01, ***p < 0.001.

### Peer rejection and internet gaming disorder

After controlling for gender, age and Internet gaming disorder at Wave 1, Peer reject at Wave 1 positively predicted Internet gaming disorder at Wave 3 (*b* = 0.04, *SE* = 0.01, *p* < 0.01).

### Mediation effect of relative deprivation

**Figure 2:**
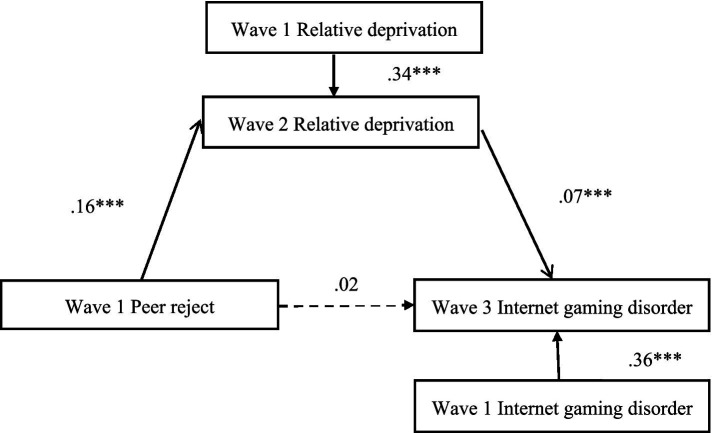
Model of the mediating role of relative deprivation between peer reject and Internet gaming disorder. Covariates including sex and age. ***p < 0.001.

The PROCESS model 4 of SPSS proposed by Hayes was used for mediation model analysis. Figure 2 displays the results of the mediation model. After controlling for gender and age, Peer reject at Wave 1 positively predicted relative deprivation at Wave 2 (*b* = 0.16, *SE* = 0.03, *p* < 0.001), Relative deprivation at Wave 2 positively predicted Internet gaming disorder at Wave 3 (*b* = 0.07, *SE* = 0.01, *p* < 0.001). The bias-corrected percentile bootstrap method showed that the mediating effect of relative deprivation at Wave 2 between peer reject at Wave 1 and Internet gaming disorder at Wave 3 was significant (indirect effect = 0.012, *SE* = 0.005, 95% CI = [0.005, 0.022]).

### Moderated mediation

**Figure 3:**
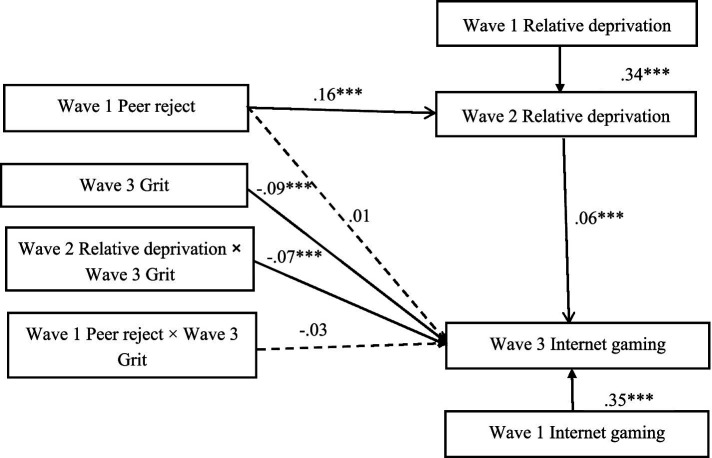
Model of the moderating role of grit on the indirect relationship between peer reject and Internet gaming disorder. Covariates including sex and age. *p < 0.05 and ***p < 0.001.

**Figure 4:**
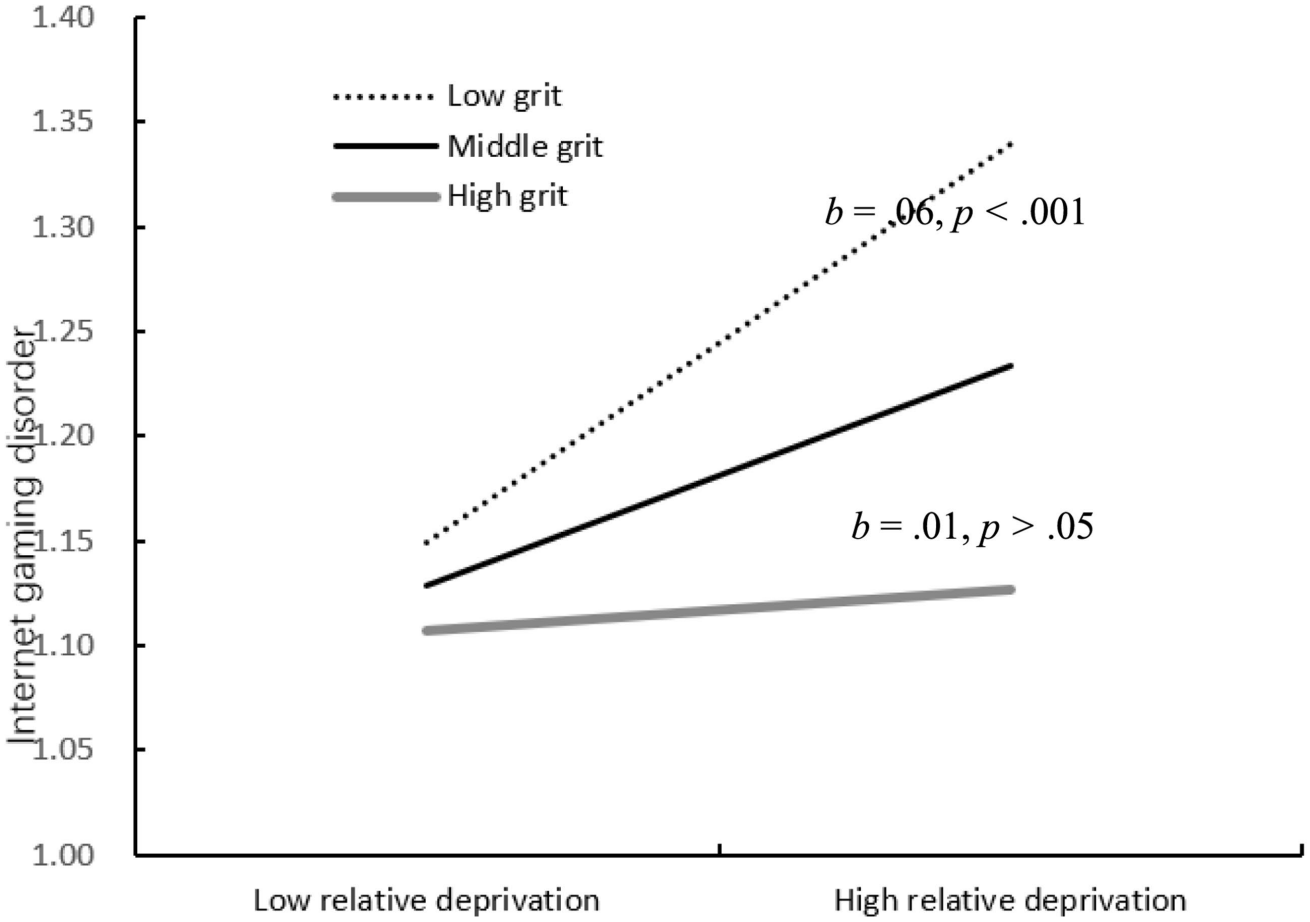
Interactive effect of relative deprivation and grit on Internet gaming disorder.

The SPSS PROCESS model15 proposed by Hayes was used for moderated mediation model analysis. Figure 3 shows the results of the moderated mediation model. After controlling for sex and age, the results indicated that indirect effect of peer reject on Internet gaming disorder through relative deprivation was moderated by grit. Specifically, Grit moderated the association between relative deprivation and Internet gaming disorder (*b* = −0.07, *SE* = 0.02, *p* < 0.001). We conducted simple slopes tests, As depicted in Figure 4, when showed low grit, the relation between relative deprivation and Internet gaming disorder was significant (*b* = 0.11, *SE* = 0.02, *p* < 0.001). However, when showed high grit, this relation was not significant (*b* = 0.01, *SE* = 0.02, *p* > 0.05).

## Discussion

The present study has found that individuals who are more severely rejected by their peers are more likely to develop subsequent Internet gaming disorder. Relative deprivation at T2 plays a complete mediating role between peer rejection at T1 and Internet gaming disorder at T3. At the same time, it was found that the personality trait of T3 grit plays a moderating role in the relationship between T2 relative deprivation and T3 Internet gaming disorder.

Firstly, there is a close relationship between T1 peer rejection and T3 Internet gaming disorder, which is consistent with previous research results. Research has also confirmed that avoiding social interactions and being addicted to the Internet can effectively reduce the pain of being excluded (Ahmed et al., 2021; Ren et al., 2021). Experiencing peer rejection makes individuals feel painful and vulnerable. Therefore, in order to escape the pain and avoid further harm, individuals will choose to participate in online games and avoid social situations (Kardefelt-Winther, 2014). After experiencing peer rejection, individuals may develop social avoidance motivation in order to avoid further harm (Ren et al., 2021). Since online games can not only satisfy the needs of the excluded to avoid interpersonal interaction, but also help alleviate the negative emotions such as worry and anxiety that occur after being excluded, it is easy for individuals to become addicted to online games and eventually lead to Internet gaming disorder. Secondly, T2 relative deprivation plays an important role in the relationship between T1 peer rejection and T3 online game addiction. In other words, the predictive effect of T1 peer rejection on T3 Internet gaming disorder cannot be achieved directly, but comes entirely from the mediating effect of T2 peer rejection. The negative interpersonal experience of peer rejection leads to the rupture of the individual’s social connection, which in turn leads to the individual’s perception of deprivation (Crosby, 1976; Walker and Pettigrew, 1984; Smith et al., 2012). Relative deprivation can lead to deviant behavior in individuals. Research has found that perceived relative deprivation can trigger negative self-experiences such as dissatisfaction and anger in individuals, leading to deviant behaviors such as addiction (Callan et al., 2015; Mishra et al., 2012). Thirdly, this study also found that the personality characteristics grit play a regulatory role in the relative deprivation of online game addiction. Specifically, individuals with high levels of grit are not easily affected by the sense of relative deprivation. Individuals with low levels of resolute levels are easily affected by relative deprivation and addicted to internet game. According to the theory of self -regulation, individuals with perseverance can be guided by the pursuit of long -term goals. In the challenge, the response strategies and management available resources are continuously adjusted to make positive self -adjustment, and the goals are achieved by paying continuous efforts and passion (Abraham and Sheeran, 2000). Therefore, individuals with high levels of grit are often able to maintain a positive mindset and emotions to cope with stress (Datu et al., 2018), and are therefore less prone to online game addiction. On the contrary, students who lack grit may feel helpless when facing stress (Usher and Pajares, 2006). It is difficult to persist in challenges and difficulties (Lucas et al., 2015), and it is easy to weaken self-efficacy (Jiang et al., 2021), making it prone to a higher risk of online game addiction.

According to the results of this study, some educational revelation can be brought. First, improving companions help reduce the risk of online game addiction. Schools can teach students’ skills to students through group counseling, psychological lectures, theme classes and other forms, and encourage students to use in daily learning life to form a good and harmonious interpersonal environment. Second, the individual adopts a positive response to facing pressure to help reduce the risk of online game addiction. Schools can carry out colorful extracurricular activities, focus on the construction of psychological counseling rooms, provide individuals with a reasonable pressure venting channel, so that individuals can use positive ways to deal with stress when facing pressure. Third, cultivating the quality of perseverance can help reduce the risk of online game addiction. Teachers and parents can use various forms to cultivate individual self-control ability and develop good living habits.

This study also has some shortcomings. First, the data of this study comes from self-reports. Affected by factors such as social desirability, the research results may be biased. Therefore, in the future, more objective data collection methods, such as experimental methods and interview methods, can be selected to further explore the problem. Second, some research has pointed out that people’s response to perceived rejection is a complex and interactive dynamic system involving cognitive, emotional, motivational and behavioral responses (Richman and Leary, 2009), future consideration may be given to examining the behavioral consequences of peer rejection from a comprehensive and dynamic developmental perspective.

## Conclusion

T1 peer rejection positively predicted T3 Internet Gaming Disorder. Relative deprivation at T2 plays a complete mediating role between peer rejection at T1 and Internet gaming disorder at T3. At the same time, it was found that the personality trait of T3 grit plays a moderating role in the relationship between T2 relative deprivation and T3 Internet gaming disorder. Individuals with high levels of grit are less likely to become addicted to Internet games even if they experience relative deprivation.

## Data Availability

The raw data supporting the conclusions of this article will be made available by the authors, without undue reservation.
